# Background parenchymal enhancement in MR and breast mammographic density in full-field digital mammography: Correlations with breast cancer risk

**DOI:** 10.1097/MD.0000000000044843

**Published:** 2025-10-03

**Authors:** Yuanyuan Ye, Wanhua Liu, Bo Xie, Qi Zhang, Chengyu Peng, Zhi Qin

**Affiliations:** a Department of Radiology, Zhongda Hospital, Medical School, Southeast University, Nanjing, China; b Department of Education, Zhongda Hospital, Medical School, Southeast University, Nanjing, China; c Department of Radiology, Center of Interventional Radiology and Vascular Surgery, Zhongda Hospital, Medical School, Southeast University, Nanjing, China.

**Keywords:** background parenchymal enhancement, breast cancer, mammography density, risk assessment

## Abstract

We attempt to reveal the correlations between breast cancer (BC) risk with mammographic density (MD) in full-field digital mammography (FFDM) and background parenchymal enhancement (BPE) in dynamic enhanced magnetic resonance imaging (MRI). 216 women who received MRI and FFDM from January 2019 to December 2020 were reviewed, among which 72 BC cases were identified histopathologically. The control was matched with the BC case in 2:1. MD in FFDM were categorized as ACR a, ACR b, ACR c, or ACR d. BPE in MR was categorized into 4 grades, minimal, mild, moderate, or marked. Logistic regression analysis was utilized to investigate the associations between BC risk with BPE and MD, resulting in the odds ratios (ORs). The review was performed with a cohort of 216 women, including 72 BC cases and 144 normal controls. Among BC cases, 64 patients were graded as ACR c or ACR d (88.9%), and 40 patients were graded as moderate or marked BPE (55.6%). The ORs for ACR c or d cases versus ACR a or b were 4.7 and 5.8 for different readers, respectively (*P* = .002). The ORs for cases exhibiting marked or moderate BPE compared to mild or minimal BPE were 5.0 and 3.3 (*P* < .001). MD and BPE categories were identified as potential risk factors for BC. Increased levels of BPE or MD are strongly predictive of BC.

## 1. Introduction

Breast cancer (BC), one of the most prevalent malignancies in women, causes an unacceptably high number of cancer-related deaths each year throughout the globe.^[1]^ While significant advancements have been achieved in the diagnostic and intervention methods for BC with the aim of decreasing mortality rates, over 500,000 fatalities are still recorded each year.^[2]^ It has been increasingly recognized that the occurrence of dense tissue in mammograms is a significant contributing factor to the development of BC.^[3]^

Mammographic density (MD) is a detection parameter that quantifies the dense area proportion in a whole breast based on the varying X-ray attenuation characteristics in disparate breast tissue compositions.^[4]^ For qualitative evaluation of MD, the American College of Radiology Breast Imaging Reporting and Data System (BI-RADS-ACR) was typically used, which is a breast imaging classification system jointly developed by experts in the field of breast radiology in the United States, and is currently the most widely used classification method. Thus, this study classified MD into 4 categories: ACR a, breast consists predominantly of fat; ACR b, dispersed regions are exhibiting fibroglandular density; ACR c, the irregular density of the breasts may mask small masses; ACR d, breast density is very high, which reducing mammography’s sensitivity.^[5]^

Early in 1976, Wolfe et al first found that the likelihood of developing BC was significantly correlated with the radiographic appearance of the breast parenchyma.^[6]^ Over the past decade, epidemiological investigations on BC’s MD have already utilized full-field digital mammography (FFDM) instead of traditional film mammography.^[7]^ Many research data have indicated that there exists a distinct correlation between the heightened susceptibility to BC and the escalated MD.^[8,9]^ Plenty of research has shown that women whose MDs are high exhibit an incremental quantity of stromal and epithelial cells, along with a reduced presence of fatty tissue. Consequently, the menace of developing BC is amplified by 4 to 6 times in their lifetime compared to individuals with low MD, indicating a larger proportion of fatty tissue.^[10]^ Nevertheless, increased MD decreases the mammography detection sensitivity for BC, particularly in females whose concentration of breast tissue was high.

In order to predict the BC odds, increased background parenchymal enhancement (BPE) at breast magnetic resonance imaging (MRI)^[11]^ was also widely utilized. BPE, known as parenchymal enhancement on MRI, can be graded into 4 qualitative groups, minimal BPE, mild BPE, moderate BPE, and significant BPE, based on BI-RADS classification.^[11,12]^ The characterizations of minimal or mild BPE exhibit bilateral, symmetric, and diffuse distribution, while moderate or marked BPE is featured by asymmetric or nondiffuse distribution.^[12]^ Brenner et al have examined the correlations between the number of fibroglandular tissue and BPE level at MRI with BC, and the findings suggested the likelihood of developing BC was significantly positively correlated with the progressive increase in fibroglandular tissue and BPE.^[13]^ However, there are few studies about the links between the likelihood of BC and both MR-BPE and FFDM-MD. In our study, women who underwent both FFDM examination and breast dynamic enhanced MR examination were included, and the relationship between BC and MR-BPE was examined and contrasted with the FFDM-MD.

## 2. Methods

### 2.1. Research methodology and participants

In the current research, a retrospective review of women who underwent both FFDM examination along breast dynamic enhanced MR examination from January 2019 to December 2020 in our hospital was conducted. The inclusion criteria were set as follows: individuals who have an interval of no more than 6 months between FFDM and breast dynamic enhanced MR; and If the subjects underwent multiple examinations from January 2011 and December 2013, the latest one was selected for analysis. During the menstrual cycle, premenopausal women were examined in the second week. The exclusion criteria included the following: patients with irregular menstruation; individuals who have received hormone replacement therapy or anti-hormone therapy 6 months before the examination; and patients who had a unilateral mastectomy, breast-conserving surgery, and breast prosthesis. The study received approval from the Research Ethics Committee of Zhongda Hospital affiliated to Southeast University (2018ZDSYLL101-P01), and written informed consent was provided for all participants.

There were 216 patients meeting the above criteria, including 143 premenopausal women and 73 postmenopausal women. Statistical power analysis was performed using GPower software to determine the minimum required sample size. With an alpha level set at 0.05, a moderate effect size (Cohen *d* = 0.3), and a desired statistical power of 0.8, the calculation indicated that 176 samples were necessary to detect significant associations between the key variables. In this study, the actual sample size of 216 exceeded this threshold, confirming that the study had sufficient power to identify the hypothesized effects. Specifically, out of the total 216 patients, 2 control subjects groups were randomly chosen and paired with each BC case individually.^[14]^ The control group consisted of 144 participants whose results of breast MRI were categorized as BI-RADS levels 1 or 2. When pairing the control group without BC with the group diagnosed with BC, subjects in the 2 groups should have the same menopause status, with age difference <5 years, and magnetic resonance (MR) examination time difference less than one year. The breast X-ray and MRI data were categorized based on the breast imaging reporting and data system (BI-RADS) guidelines established by the American College of Radiology (ACR).^[11]^ Patients were required to undergo biopsy if the MR results indicated lesions defined as BI-RADS 4 or 5. Among these 216 patients, 72 cases were confirmed as BC by pathology records. A final cohort of examinations was reviewed in 216 women, including 72 BC cases and 144 normal control individuals.

### 2.2. Examinations

In the second week of the menstrual cycle, the examinations were conducted. FFDM examination was conducted on a Senographe 2000D (GE Healthcare, Chicago) FFDM, including the routine mediolateral oblique view and cranio-caudal view. MRI scanning was conducted on a Siemens MAGNETOM Verio 3.0T Scanner (Siemens, Germany) with the patient prone using an 8-channel phased array coil. A 3D FLASH sequence with shared views is followed by a T1-weighted sequence with fat suppression. (conditions: repetition time = 4.76 ms, echo time = 1.66 ms, field of view = 384 × 384 mm, section thickness = 1.2 mm) was included in the imaging process. Scanning lasted consecutively for 9 minutes 34 seconds and 9 time phases were collected, with each dynamic phase collecting 59 seconds. The administration of gadolinium-diethylenetriamine pentaacetic acid (Gd-DTPA) as a contrast agent involved the rapid injection of 0.1 mmol/kg body weight, at a speed of 2.0 mL/s, followed by the infusion of 20 mL normal saline using the same injection rate. The dynamic enhanced MR scanning was started immediately after the injections were finished. The first phase after enhancement was selected to perform subtraction with the mask, and contrast enhanced maximum intensity projection (MIP) images were reconstructed.

### 2.3. Imaging analysis

**Figure 1.**
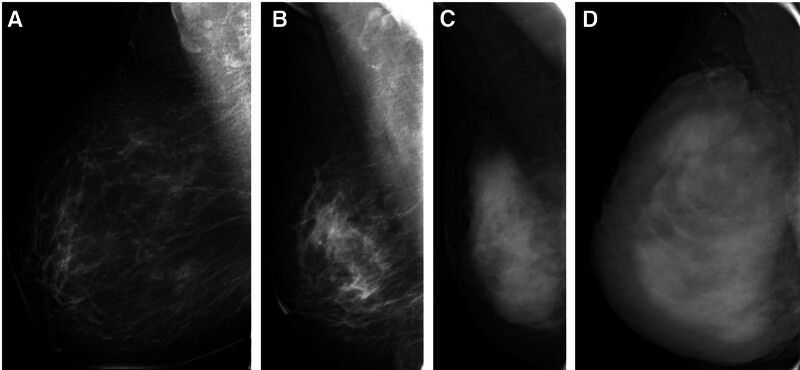
Different categories of breast MD in FFDM according to the ACR Breast BI-RADS. (A) ACR a; (B) ACR b; (C) ACR c; (D) ACR d. ACR = American College of Radiology, BI-RADS = breast imaging reporting and data system, FFDM = full-field digital mammography, MD = mammographic density.

**Figure 2.**
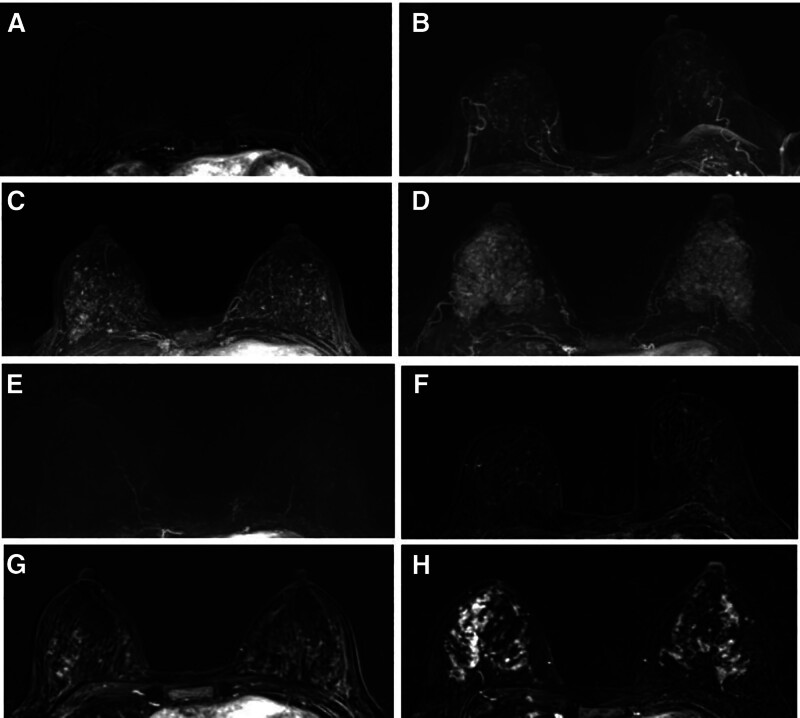
Different categories of BPE images according to MRI scanning. Contrast enhanced MIP subtraction images: (A) minimal; (B) mild; (C) moderate; (D) marked. Subtraction with the mask: (E) minimal; (F) mild; (G) moderate; (H) marked. BPE = background parenchymal enhancement, MIP = maximum intensity projection, MRI = magnetic resonance imaging.

All image data were reviewed separately by 2 experienced breast imaging radiologists (Reader 1 has 7 years of comprehensive experience with breast MRI; Reader 2 has 4 years of experience). It is noted that the MRI data from BC patients were merged with that of the normal controls, and the radiologists analyzing imaging features (including BPE and MD) were blinded to patient outcomes and clinical diagnoses. Meanwhile, the BI-RADS classification criteria for breast MD were utilized to grade it, including ACR a, ACR b, ACR c, and ACR d (Fig. 1). The BPE of the whole breast parenchyma was categorized into 4 categories: minimal (<25% glandular tissue enhancement, nearly all fat), mild (from 25–50% glandular tissue enhancement), moderate (from 50–75% glandular tissue enhancement), and marked (more than 75 glandular tissue enhancement) (Fig. 2).

### 2.4. Statistical analysis

SPSS 22.0 software (SPSS, Inc., Chicago) was utilized to conduct all statistical analyses, and *P* < .05 was determined as statistically significant. The odds ratios (ORs) and 95% confidential intervals were calculated through conditional logistic regression analysis to identify the potential risk factors associated with BC. FFDM-MD and MR-BPE of the BC group and healthy control group were assessed by the 2 readers, and the assessment results of the more experienced reader 1 were stated. The consistency of results from the 2 readers was determined by weighted Kappa analysis according to the κ values: The consistency levels are defined as 6 levels with κ values difference of approximately 0.2 for each level, specifically, poor (κ < 0.00), mild (κ: 0.00–0.20), fair (κ: 0.21–0.40), moderate (κ: 0.41–0.60), basic (κ: 0.61–0.80), and complete (κ: 0.81–1.00).

## 3. Results

### 3.1. Patients grouping

The average age of the 72 participants in the BC group was 47.7 years (47.7 ± 9.4), and their ages ranged from 26 to 80. The average age of the 144 normal control subjects, who ranged in age from 24 to 85 years, was 48.1 years (48.1 ± 9.4). Based on the assessment results of reader 1, among all the enrolled 216 individuals, 166 patients were graded as ACR c or ACR d (76.9%, 166/216). The percentage of patients graded as minimal or mild BPE was 67.6% (146/216), which was relatively higher than that of patients graded as moderate or marked BPE (32.4%, 70/216). Besides, among 72 BC cases, 64 patients were graded as ACR c or ACR d (sensitivity = 88.9%, 64/72), and 40 patients were graded as moderate or marked BPE (sensitivity = 55.6%, 40/72). Among the 144 normal controls, 42 individuals were graded as ACR a or ACR b (specificity = 29.2%, 42/144), and 114 individuals were graded as minimal or mild BPE (specificity = 79.2%, 114/144).

### 3.2. Logistic regression analysis of MR-BPE with BC

Logistic regression analysis revealed that the MR-BPE level exhibited a significant independent BC risk factor based on the assessing results from both reader 1 (*P* < .001) and reader 2 (*P* < .001) (Table 1). Compared with those exhibiting mild BPE or minimal BPE cases, those exhibiting marked BPE or moderate BPE had larger risks of BC according to both the assessing results from both reader 1 (OR = 5.0, 95% CI: 2.5–10.0) and reader 2 (OR = 3.3, 95% CI: 1.7–6.2).

**Table 1:** Correlations of background parenchymal enhancement (BPE) levels with odds of breast cancer by logistic regression analysis.

BPE levels	Breast cancer group	Normal control group	OR (95% CI)	*P*
Reader 1				
Minimal or mild	32/72 (44.4%)	114/144 (79.2%)	1.0	<.001
Moderate or marked	40/72 (55.6%)	30/144 (20.8%)	5.0 (2.5, 10.0)	
Reader 2				
Minimal or mild	35/72 (48.6%)	107/144 (74.3%)	1.0	<.001
Moderate or marked	37/72 (51.4%)	37/144 (25.7%)	3.3 (1.7, 6.2)	
Premenopausal subgroup analysis				
Reader 1				
Minimal or mild	19/47 (40.4%)	71/96 (74.0%)	1.0	.002
Moderate or marked	28/47 (59.6%)	25/96 (26.0%)	3.3 (1.5, 7.1)	
Reader 2				
Minimal or mild	20/47 (42.6%)	66/96 (68.8%)	1.0	.016
Moderate or marked	27/47 (57.4%)	30/96 (31.2%)	2.6 (1.2, 5.7)	
Postmenopausal subgroup analysis				
Reader 1				
Minimal or mild	13/25 (52.0%)	43/48 (89.6%)	1.0	.038
Moderate or marked	12/25 (48.0%)	5/48 (10.4%)	9.4 (1.1, 78.0)	
Reader 2				
Minimal or mild	15/25 (60.0%)	41/48 (85.4%)	1.0	.032
Moderate or marked	10/25 (40.0%)	7/48 (14.6%)	1.9 (0.5, 7.2)	

BPE = background parenchymal enhancement, CI = confidential interval, OR = odds ratio.

MR-BPE level was also a significant independent BC risk factor in premenopausal subgroup (reader 1: *P* = .002; reader 2: *P* = .016) and postmenopausal subgroup (reader 1: *P* = .038) (Table 1). Moderate or marked BPE cases had obviously greater risk of BC compared to minimal or mild cases of BPE in premenopausal subgroup (reader 1: OR = 3.3, 95% CI: 1.5–7.1; reader 2: OR = 2.6, 95% CI: 1.2–5.7) and postmenopausal subgroup (reader 1: OR = 9.4, 95% CI: 1.1–78.0).

### 3.3. Logistic regression analysis of FFDM-MD with BC

FFDM-MD category was revealed to be a significant independent BC risk factor we found according to the logistic regression analysis (reader 1: *P* = .002; reader 2: *P* = .002) (Table 2). Cases with ACR c or ACR d had higher risks of BC versus those exhibiting ACR a or ACR b according to both the assessing results from both reader 1 (OR = 4.7, 95% CI: 1.7–12.7) and reader 2 (OR = 5.8, 95% CI: 1.9–17.0).

**Table 2:** Correlations of breast mammographic density (MD) category with odds of breast cancer by logistic regression analysis.

MD category	Breast cancer group	Normal control group	OR (95% CI)	*P*
Reader 1				
ACR a or ACR b	8/72 (11.1%)	42/144 (29.2%)	1.0	.002
ACR c or ACR d	64/72 (88.9%)	102/144 (70.8%)	4.7 (1.7, 12.7)	
Reader 2				
ACR a or ACR b	9/72 (12.5%)	45/144 (31.2%)	1.0	.002
ACR c or ACR d	63/72 (87.5%)	99/144 (68.8%)	5.8 (1.9, 17.0)	
Premenopausal subgroup analysis				
Reader 1				
ACR a or ACR b	1/47 (2.1%)	17/96 (17.7%)	1.0	.039
ACR c or ACR d	46/47 (97.9%)	79/96 (82.3%)	8.9 (1.1, 70.7)	
Reader 2				
ACR a or ACR b	1/47 (2.1%)	18/96 (18.8%)	1.0	.046
ACR c or ACR d	46/47 (97.9%)	78/96 (81.2%)	8.4 (1.0, 67.2)	
Postmenopausal subgroup analysis				
Reader 1				
ACR a or ACR b	7/25 (28.0%)	25/48 (52.1%)	1.0	.036
ACR c or ACR d	18/25 (72.0%)	23/48 (47.9%)	4.2 (1.1, 15.8)	
Reader 2				
ACR a or ACR b	8/25 (32.0%)	27/48 (56.2%)	1.0	.023
ACR c or ACR d	17/25 (68.0%)	21/48 (43.8%)	6.1 (1.3, 28.9)	

ACR = American College of Radiology, CI = confidential interval, MD = mammographic density, OR = odds ratio.

FFDM-MD was also a significant independent risk factor of BC in the premenopausal subgroup (reader 1: *P* = .039; reader 2: *P* = .046) and postmenopausal subgroup (reader 1: *P* = .036; reader 2: *P* = .023) (Table 2). ACR c or ACR d BPE cases had obviously higher risk of BC than ACR a or ACR b cases in premenopausal subgroup (reader 1: OR = 8.9, 95% CI: 1.1–70.7; reader 2: OR = 8.4, 95% CI: 1.0–67.2) and postmenopausal subgroup (reader 1: OR = 4.2, 95% CI: 1.1–15.8; reader 2: OR = 6.1, 95% CI: 1.3–28.9).

### 3.4. Comparison of MR-BPE and FFDM-MD with odds of BC

In premenopausal subgroup analysis, increased FFDM-MD showed higher BC risk when compared with the MR-BPE levels, based on the assessing results from both reader 1 (FFDM-MD: OR = 8.9, 95% CI: 1.1–70.7; MR-BPE: OR = 3.3, 95% CI: 1.5–7.1) and reader 2 (FFDM-MD: OR = 8.4, 95% CI: 1.0–67.2; MR-BPE: OR = 2.6, 95% CI: 1.2–5.7) (Table 3). Weighted Kappa analysis results indicated that there were good consistencies between reader 1 and reader 2 concerning the assessment results of MR-BPE (κ = 0.875) and FFDM-MD (κ = 0.823).

**Table 3:** Comparison of the correlations of BPE levels and MD category with odds of breast cancer.

Comparison analysis	BPE levels OR (95% CI)	MD categories OR (95% CI)
Reader 1	5.0 (2.5, 10.0)	4.7 (1.7, 12.7)
Reader 2	3.3 (1.7, 6.2)	5.8 (1.9,17.0)
Premenopausal subgroup analysis		
Reader 1	3.3 (1.5, 7.1)	8.9 (1.1, 70.7)
Reader 2	2.6 (1.2, 5.7)	8.4 (1.0, 67.2)
Postmenopausal subgroup analysis		
Reader 1	9.4 (1.1, 78.0)	4.2 (1.1, 15.8)
Reader 2	1.9 (0.5, 7.2)	6.1 (1.3, 28.9)

BPE = background parenchymal enhancement, CI = confidential interval, MD = mammographic density, OR = odds ratio.

## 4. Discussion

BC is one of the malignant tumors that seriously threaten women’s health.^[9]^ It is of great significance to explore its related risk factors for the prevention and early intervention of BC.^[8,15]^ This study concluded that in premenopausal or postmenopausal women, the BC risk of ACR c or ACR d cases was significantly higher than that of ACR a or ACR b cases, the BC risk of moderate or severe BPE cases was significantly higher than that of mild or mild BPE cases, and in premenopausal women, elevated MD levels had a higher BC risk than elevated BPE levels, which provided valuable clues for further understanding the pathogenesis of BC.

From the perspective of breast imaging, ACR classification reflects the different characteristics of breast tissue.^[16–18]^ ACR c and ACR d cases may indicate the presence of more fibroglandular tissue, structural disorders, or other potential pathological changes in breast tissue. These changes may affect the microenvironment of breast cells and promote the occurrence and development of tumors.^[19]^ For example, complex fibroglandular structures may lead to abnormal intercellular signaling or be more prone to abnormal proliferation under hormone action. In contrast, breast tissue in ACR a or ACR b cases is relatively normal, with better regulation of cell growth and proliferation, thereby reducing the risk of BC. This suggests that in clinical practice, for premenopausal and postmenopausal women with ACR c or ACR d cases, screening and monitoring of BC should be strengthened, and shorter screening intervals or more sensitive detection methods, such as breast MRI, can be considered to detect possible tumor lesions early.

Meanwhile, the association between the degree of BPE and the risk of BC cannot be ignored, and BPE has emerged as a potential imaging biomarker in contrast-enhanced breast imaging^[20]^ In line with previous study, we discovered that mild cases of BPE have a lower risk of BC due to the relatively mild pathological and physiological changes in their breast tissue, which provides a basis for the development of personalized BC prevention strategies.^[21]^ However, moderate or severe BPE may reflect vascular proliferation, inflammatory response, or increased hormone sensitivity in breast tissue. Vascular proliferation can provide abundant nutrients for tumor growth, while inflammatory reactions may promote tumor cell proliferation, invasion, and metastasis by releasing cytokines and other factors.^[22]^ Compared with mild BPE cases, we discovered the risk of BC in moderate or severe BPE cases increases significantly. the risk of BC in moderate or severe BPE cases increases significantly. Therefore, it is essential to closely monitor breast health status and perform regular breast examinations.^[23,24]^ Interestingly, researchers noted that the hormone levels of postmenopausal women may further modulate the effects of BPE on breast tissue. Specifically, BPE has been demonstrated to correlate with both endogenous and exogenous hormone exposure, with a significant positive association observed between BPE and serum estradiol levels in postmenopausal women.^[25]^ This finding suggests that BPE could serve as a reliable imaging biomarker for evaluating breast hormonal exposure, holding promise as an indicator for the further stratification of high-risk women undergoing BC screening via MRI.^[26]^

Likewise, the level of MD may be closely related to the density and cellular composition of breast tissue. A higher level of MD may suggest an increased proportion of epithelial cells and stromal cells in breast tissue, more active cell proliferation, and possibly more opportunities for gene mutations. Prior study pointed that estrogen can directly act on breast epithelial cells, promoting their proliferation.^[27]^ In premenopausal women, estrogen levels fluctuate significantly during the menstrual cycle, which leads to more pronounced breast cell proliferation accompanied by elevated MD, thereby increasing their risk of BC.^[28]^ Additionally, several studies have indicated that elevated MD is associated with a higher risk of BC than elevated BPE.^[29–31]^ In this study, we observed that as MD increases, breast tissue becomes more susceptible to carcinogenesis under estrogen stimulation, which indicates that MD levels should be regarded as an important indicator and given significant attention in BC risk assessment among premenopausal women. Nevertheless, it is crucial to emphasize that the utilization of MD as a sole risk indicator results in an elevated incidence of false positives.^[32]^ Consequently, the integration of multi-factor joint analysis is imperative to enhance the precision of risk stratification.

However, this study also has certain limitations. First, as a retrospective analysis, it is based on data from a single center with a relatively small sample size. Second, the research findings are derived solely from existing case data and qualitative detection indicators, which may restrict the generalizability of the conclusions. Therefore, future studies involving multi-center cohorts with diverse genetic backgrounds are warranted to validate these results. Additionally, quantitative analysis and multi-factor investigations into potential risk factors of BC should be conducted to more comprehensively and accurately assess the risk of BC in premenopausal and postmenopausal women. At the same time, exploring the interaction mechanisms among these risk factors is also necessary to provide a solid theoretical basis for developing more effective strategies for BC prevention and treatment.

## 5. Conclusion

In conclusion, ACR c or ACR d cases had obviously higher risks for BC than ACR a or ACR b cases in either premenopausal or postmenopausal women. Moderate or marked BPE cases had obviously higher risk for BC than minimal or mild BPE cases. Increased MD levels have higher BC risk than increased BPE levels in premenopausal women.

## Author contributions

**Conceptualization:** Yuanyuan Ye.

**Data curation:** Wanhua Liu, Bo Xie.

**Formal analysis:** Wanhua Liu, Bo Xie, Zhi Qin.

**Investigation:** Qi Zhang, Chengyu Peng.

**Methodology:** Qi Zhang, Chengyu Peng.

**Writing – original draft:** Yuanyuan Ye.

**Writing – review & editing:** Yuanyuan Ye.
